# ON AN ALLEGED TRUTH/FALSITY ASYMMETRY IN CONTEXT SHIFTING EXPERIMENTS

**DOI:** 10.1111/j.1467-9213.2012.00059.x

**Published:** 2012-04-10

**Authors:** Nat Hansen

**Affiliations:** Umeå University

## Abstract

Keith DeRose has argued that context shifting experiments should be designed in a specific way in order to accommodate what he calls a ‘truth/falsity asymmetry’. I explain and critique DeRose's reasons for proposing this modification to contextualist methodology, drawing on recent experimental studies of DeRose's bank cases as well as experimental findings about the verification of affirmative and negative statements. While DeRose's arguments for his particular modification to contextualist methodology fail, the lesson of his proposal is that there is good reason to pay close attention to several subtle aspects of the design of context shifting experiments.

## I. Context Shifting Experiments

Language contains expressions that shift their content in different contexts. There is a sprawling debate that concerns which expressions are context sensitive and how best to explain context sensitivity. Sometimes the debate concerns whether philosophically significant expressions like ‘know’ or ‘wrong’ are context-sensitive, and the context-sensitivity of these expressions is alleged to have important ramifications for classic problems in epistemology, ethics and other central areas of philosophy.

Various techniques are employed to show that particular expressions are context sensitive, but perhaps the most widely used involves constructing *context shifting arguments*.^1^ A context shifting argument consists of a *context shifting experiment*, which elicits intuitions about utterances containing expression *e* in different imagined contexts, and an argument that the best way to explain the intuitions generated in response to the experiment involves semantic features of *e*.

Context shifting experiments typically have the following form: they describe two different contexts C1 and C2 in which one is meant to evaluate the truth or falsity (or some other semantically or pragmatically relevant property) of what is said by an utterance of a target sentence *TS*.^2^ In each of the two contexts, there is a state of affairs *SoA* that remains fixed. The *SoA* is in some intuitive sense what the utterances of the sentence *TS* are about. The two contexts C1 and C2 differ in certain background conditions, usually in terms of the interests of conversational participants in the context. The person imagining the two contexts is supposed to feel an intuitive change in the truth value (or some other semantically relevant property) of what the speaker says in uttering *TS* in the two contexts.

As an illustration of the structure of context shifting experiments, consider the following example, due to Charles Travis, involving the leaves of a Japanese maple that have been painted green, a context (C1) in which someone is decorating, a second context (C2) in which a botanist is looking for leaves to use in a study of green leaf chemistry, and two utterances of the target sentence ‘The leaves are green’, one in each context:

A story. Pia's Japanese maple is full of russet leaves. [She paints them green ‘for a decoration’.^3^ Returning, she reports, ‘That's better. The leaves are green now’. She speaks truth. A botanist friend then phones, seeking green leaves for a study of green-leaf chemistry. ‘The leaves (on my tree) are green’, Pia says. ‘You can have those’. But now Pia speaks falsehood.^4^

There are several competing explanations of Travis's intuitions about the *painted leaves* case (the intuitions are presented schematically in Table 1).^5^

**Table 1:** Travis's Intuitions about the *Painted Leaves* Case

	C1	C2
	*Decorator*	*Botanist*
‘The leaves are green’	TRUE	FALSE

While there has been an enormous amount of debate about how best to explain the intuitions elicited by context shifting experiments like Travis's painted leaves case, until recently there has been comparatively little discussion of the methods by which the intuitions are elicited.^6^ One prominent exception to the neglect of how intuitions are elicited by context shifting experiments is Keith DeRose.^7^

## II. DeRose and Experimental Design

DeRose has argued that subtle features of the design of context shifting experiments can affect the intuitions that they generate. Rather than following the standard procedure (exemplified by Travis's painted leaves case) according to which one aims to generate changing intuitions about the semantic properties of a target sentence *TS*, DeRose argues that a better procedure is to construct a pair of contexts in which *TS* and the *contradictory* of *TS* are both intuitively *true* of a given state of affairs *SoA*. If it is possible to construct such a pair of contexts, that is supposed to be evidence that *TS* and its contradictory are context sensitive.

DeRose's well known *bank* case is designed in accordance with this recommendation. We are first asked to evaluate the truth value of an utterance of ‘I know the bank will be open on Saturday’ in a ‘low standard’ context (Case A), and then we are asked to evaluate the truth value of an utterance of ‘I don’t know the bank will be open on Saturday’ in a ‘high standard’ context (Case B):

Bank Case A. My wife and I are driving home on a Friday afternoon. We plan to stop at the bank on the way home to deposit our paychecks. But as we drive past the bank, we notice that the lines inside are very long, as they often are on Friday afternoons. Although we generally like to deposit our paychecks as soon as possible, it is not especially important in this case that they be deposited right away, so I suggest that we drive straight home and deposit our paychecks on Saturday morning. My wife says, ‘Maybe the bank won’t be open tomorrow. Lots of banks are closed on Saturdays'. I reply, ‘No, I know it’ll be open. I was just there two weeks ago on Saturday. It's open until noon.' [The bank is open on Saturday.]

Bank Case B. My wife and I drive past the bank on a Friday afternoon, as in Case A, and notice the long lines. I again suggest that we deposit our paychecks on Saturday morning, explaining that I was at the bank on Saturday morning only two weeks ago and discovered that it was open until noon. But in this case, we have just written a very large and very important check. If our paychecks are not deposited into our checking account before Monday morning, the important check we wrote will bounce, leaving us in a *very* bad situation. And, of course, the bank is not open on Sunday. My wife reminds me of these facts. She then says, ‘Banks do change their hours. Do you know the bank will be open tomorrow?’ Remaining as confident as I was before that the bank will be open then, still, I reply, ‘Well, no, I don’t know. I’d better go in and make sure'. [The bank is open on Saturday.]^8^

[…] It seems to me that (1) when I claim to know that the bank will be open on Saturday in Case A, I am saying something true. But it also seems that (2) I am saying something true in Case B when I say that I *don't* know that the bank will be open on Saturday.^9^

In support of this way of designing context shifting experiments, DeRose argues that it generates ‘mutually reinforcing strands of evidence’ in favour of the context sensitivity of what is claimed in knowledge ascriptions in a way that the standard design does not. The first strand of evidence consists of the truth value intuitions DeRose reports (given in Table 2).

**Table 2:** DeRose's Truth-Value Intuitions about the *Bank* Case

	Case A	Case B
	*Low standard*	*High standard*
‘I know the bank will be open on Saturday’	TRUE	
‘I don’t know the bank will be open on Saturday’		TRUE

The second strand of evidence consists in the ‘facts that [DeRose's] cases display how speakers in fact, and with propriety, use the claims in question’.^10^ That is, DeRose's version of the context shifting experiments presents two examples of *conversationally appropriate* uses of language, whereas standard context shifting experiments (like the painted leaves case) involve one conversationally appropriate and one conversationally inappropriate use of language (see Tables 3 and 4):

**Table 3:** DeRose's Appropriateness Intuitions about the *Bank* Case

	Case A	Case B
	*Low standard*	*High standard*
‘I know the bank will be open on Saturday’	TRUE + appropriate	
‘I don’t know the bank will be open on Saturday’		TRUE + appropriate

**Table 4:** Appropriateness Intuitions about the *Painted Leaves* Case

	C1	C2
	*Decorator*	*Botanist*
‘The leaves are green’	TRUE + appropriate	FALSE + inappropriate

Pia's assertion in the botanist context appears inappropriate because it seems intentionally misleading. Pia knows that the leaves are not naturally green, and (though it isn't explicitly stated in the story of the painted leaves) it seems she would know that it is their natural colour that her botanist friend is asking about when she asks whether Pia has any green leaves.

According to DeRose, the second strand of evidence reinforces the first by providing support for the idea that both claims in the experiment are true because there is a ‘general [reasonable] presumption’ that a natural and appropriate description of a situation will be a true description:

But I think the reason [the second strand of evidence] helps in supporting the claim that what one's imagined speaker is saying is true is that it engages the general presumption that where speakers are not basing their claims on some false beliefs they have about underlying matters of fact, how they naturally and appropriately describe a situation, especially by means of very common words, will be a true description.^11^

According to DeRose, his approach is supposed to be more reliable than the standard procedure for constructing context shifting experiments because the standard procedure involves finding certain claims *conversationally inappropriate*, and the conversational inappropriateness of a claim ‘cannot be used to buttress the intuition that the claim is false with the same security as one can use the appropriateness of a claim to reinforce the intuition that the claim is true’. DeRose calls this difference a ‘truth/falsity asymmetry’.^12^ He explains his reasons for believing in the asymmetry in the following passage:

[W]hen a speaker makes a claim that is, and is from her own point of view, false, her claim will be improper as well as false. But this impropriety cannot be used to buttress the intuition that the claim is false with the same security as one can use the appropriateness of a claim to reinforce the intuition that the claim is true, because there is not nearly as strong a presumption that inappropriate claims are false as there is that appropriate claims are true. As David Lewis points out, ‘There are ever so many reasons why it might be inappropriate to say something true. It might be irrelevant to the conversation, it might convey a false hint, it might be known already to all concerned’.^13^ And though Lewis does not go on to say so, the comparative point he is making depends on, and he therefore implies, that it is not nearly as likely that an appropriately made claim will be false. And that implied point seems right. For it seems that except where we engage in special practices of misdirection, like irony or hyperbole, we should seek to avoid asserting falsehoods, and we will thus be speaking improperly if we assert what is, from our own point of view, false.^14^

The conclusion of DeRose's argument, that the conversational inappropriateness of a claim *q* cannot be used to buttress the intuition that *q* is false with the same security that the conversational appropriateness of a claim *p* can be used to buttress the intuition that *p* is true, is a reason for believing there is a ‘truth/falsity asymmetry’ in context shifting experiments. According to DeRose, the intuitions of appropriateness elicited by the design of DeRose's bank case (see Table 3) buttress the intuitions of truth value DeRose reports (see Table 2), whereas the intuition of inappropriateness in C2 (the botanist context) of Travis's painted leaves case (see Table 4) does not buttress the intuition that what is claimed in C2 is false. That idea speaks in favour of DeRose's design and against the traditional design of context shifting experiments.

DeRose has invoked the ‘truth/falsity asymmetry’ in his response to recent experimental results that seem to provide empirical evidence that purport to challenge contextualist theories.^15^ DeRose's response to the challenge posed by recent experimental findings offers a second reason in favour of his claim that there is a truth/falsity asymmetry in context shifting experiments.

## III. DeRose and Surveys about ‘Know’

Wesley Buckwalter surveyed responses to versions of DeRose's bank case that followed the standard design for context shifting experiments in asking subjects to report intuitions about a single affirmative sentence uttered in both contexts, instead of DeRose's version.^16^ Rather than asking subjects for absolute truth-value judgements, Buckwalter asked subjects to perform the following task with regard to one bank context:

On a scale of 1 to 5, circle how much you agree or disagree that [DeRose's] assertion, ‘I know the bank will be open on Saturday’ is true.^17^

Buckwalter's survey found no statistically significant difference between subjects' agreement with the the assertion when it concerned low-stakes and high-stakes contexts, or when it concerned high or low ‘standards’ contexts in which the salience of possibilities of error was changed. Buckwalter takes these results to call the empirical foundation of contextualism into question, since it seems that contextualism about knowledge ascriptions would predict that there should be a significant difference between the degree of ordinary speakers' agreement with the idea that an assertion of ‘I know the bank will be open on Saturday’ is true in the contexts Buckwalter tested.^18^

DeRose replies to Buckwalter's findings by criticising his survey for ignoring the truth/falsity asymmetry and one of the ‘strands' of support that DeRose attributes to his design, namely the claim that intuitions of *appropriateness* support intuitions of truth in a way that intuitions of *inappropriateness* do not.^19^ When the experimental set up is changed from DeRose's recommended design to the more traditional contextualist design employed by Buckwalter, DeRose says:

It would be no great surprise to me if the intuition I appeal to dissipates when what I think it is partly based on is removed, as it is when Buckwalter changes the cases in the way we are currently considering.

In DeRose's reply, he quotes a passage from his *The Case for Contextualism* (pp. 71–2) that gives an additional reason in favour of his specific design and against the design of the surveys conducted by Buckwalter:

It may be more difficult than one might think to devise a case that is otherwise suitable to the argument and in which it really does strike us as intuitively clear that the positive ascription in the [high-stakes context] is false, because there is pressure on us as interpreters of the ascription to understand it as having a content that makes it true, due to the operation of what David Lewis calls a ‘rule of accommodation’.

DeRose continues:

To change my argument for contextualism so that what was an important and powerful aide to it—accommodation—is instead a force working against it is potentially to damage it quite significantly.

DeRose therefore has two arguments in favour of his particular design and the truth/falsity asymmetry:

1. The conversational inappopriateness of a claim *q* cannot be used to buttress the intuition that *q* is false with the same security that the conversational appropriateness of a claim *p* can be used to buttress the intuition that *p* is true.
2. There is a rule of accommodation that puts pressure on us as interpreters to understand what a speaker says as having a content that makes it true. It is therefore no surprise that an experiment that is supposed to test for contextualist intuitions but which requires subjects have to have the intuition that what a speaker says is true in one context and *false* in another will not produce results that favour contextualism.

I will evaluate both of these arguments in the following section.

## IV. Problems with DeRose's Arguments

Consider again the first argument DeRose gives for the existence of the truth/falsity asymmetry, which has the following structure:

*Lewis's point*: There is not a reasonable presumption that a conversationally inappropriate claim is false, because ‘there are ever so many reasons why it might be inappropriate to say something true’.

*DeRose's point*: There is a reasonable presumption that a conversationally appropriate claim is true.

*Conditional assumption*: If there is a reasonable presumption that a conversationally appropriate claim is true, and there is not a reasonable presumption that a conversationally inappropriate claim is false, then the conversational inappropriateness of a claim *q* cannot be used to buttress the claim that *q* is false with the same security that the conversational appropriateness of a claim *p* can be used to buttress the intuition that *p* is true.

*Conclusion*: So the conversational inappropriateness of a claim *q* cannot be used to buttress the intuition that *q* is false with the same security that the conversational appropriateness of a claim *p* can be used to buttress the intuition that *p* is true.

*Lewis's point* is surely correct. What about *DeRose's point*? Should we think that the inference from conversational appropriateness to truth is any more secure than the obviously suspect inference from conversational inappropriateness to falsehood?

It is far from obvious that *DeRose's point* is true, because its plausibility depends on a controversial rejection of a particular conception of *what is said* in making an utterance. In order to explain the worry about *DeRose's point*, some background is necessary.

How what is said by an utterance is determined by the interaction of the linguistic meaning of the sentence uttered and the context in which it is uttered is a subject of intense debate. One way of framing the debate is in terms of whether the contribution that context makes to what is said by an utterance is ‘linguistically controlled’ by the meaning of the uttered sentence. Contextual effects that are linguistically controlled are limited to the assignment of referents to indexicals and values to free variables. ‘Minimalists’ about what is said argue that the effects of context on what is said must be so controlled, and otherwise context only affects what is conveyed or implicated by the utterance. Advocates of ‘free enrichment’ of what is said, on the other hand, deny that the contribution that context makes to what is said is controlled by the linguistic meaning of the uttered sentence, and instead context can ‘enrich or otherwise modify’ what is said by an utterance so that the utterance makes sense in context.^20^

The following example, from Kent Bach, makes the difference between minimalist and free enrichment views of what is said more concrete. Consider (1), uttered by a parent as a way of consoling her child who has just suffered a minor cut on her hand:

1. You are not going to die.^21^

An advocate of free enrichment would point out that a speaker who uttered (1) in the circumstances described would say something that was truth conditionally equivalent to *You are not going to die from that cut*—that is, she would say something true in the circumstances described.^22^ A minimalist, on the other hand, would hold that what is said by an utterance of (1) is determined exclusively by the meaning of the words of the uttered sentence, the way those meanings are put together, and forms of linguistically controlled context dependence (like the assignment of the addressee as the referent of ‘you’). On that understanding of what the speaker says, (1) would be false in the circumstances described, because, like all mortals, the child is going to die eventually. Nothing in the meaning of (1) dictates that context should supply the additional material *from that cut*.

This is not the place to enter into the debate over whether a minimalist view or a free enrichment view is the best way to understand the notion of what is said by an utterance. But the choice between minimalist and free enrichment views is relevant to assessing *DeRose's point*, because its plausibility depends on rejecting minimalism about what is said. A minimalist would find many examples of appropriately made utterances that nevertheless say things that are ‘false from [one's] own point of view’. For example, consider (2–8):

(2) Mary arrived at three o'clock.^23^
(3) I live in Paris.^24^
(4) I haven't had dinner.
(5) Holland is flat.
(6) I must run to the bank before it closes.
(7) I need a Kleenex.^25^
(8) A vacuum has been established in that can of peanuts.^26^

A minimalist would maintain that in the right circumstances, a speaker can believe that utterances of (2–8) say something *false* and yet that they are still conversationally appropriate, even though none of (2–8) involves hyperbole or irony. For example, imagine (2) uttered when Mary arrived at five minutes to three and nothing important turns on a precise specification of arrival time; imagine (3) uttered when the speaker, who lives in Issy-les-Moulineaux, ‘a block away from the city limits of Paris’, is having a casual conversation with someone unfamiliar with the details of Paris suburbs at a party in London (Sperber and Wilson, p. 163); imagine (4) uttered as a response to an invitation to eat dinner, even though the speaker had dinner the night before; (5) can be appropriate to say even when one knows that ‘Holland is not a plane surface’ (Wilson and Sperber, p. 592); (6) can be appropriately uttered even if the speaker must only walk quickly to the bank; (7) may be uttered appropriately by someone who is indifferent to the particular brand of tissue she needs; and even though there isn't a vacuum in the relevant can of peanuts, (8) can be appropriately uttered as a succinct way of conveying that ‘the interior of [that can], surrounding the nuts, is near enough to being a vacuum for the purposes at hand’ (Unger, p. 52). It appears to be common, from a minimalist point of view, even when not employing hyperbole or irony, that one can speak appropriately even when what is said is, from one's own point of view, false.

An advocate of free enrichment would disagree with the minimalist reading of (2–8) as *appropriate but false* in the circumstances described. For example, free enrichment would allow an assertion of (4) in the circumstances described to express the (true) proposition *I haven't had dinner tonight*.

It's not clear whether DeRose embraces free enrichment.^27^ But he cannot embrace minimalism while holding on to his presumption of truth for appropriate claims, since (2–8) are examples of the many appropriate but false claims that minimalism allows. Whether a minimalist or free enrichment view of what is said is correct is an extremely controversial issue, so the presumption that appropriate claims are true, which holds only if minimalism about what is said is rejected, should be equally controversial, and DeRose isn't entitled to rely on the presumption to provide independent support for his particular design of context shifting experiments.^28^

It is therefore not at all obvious that an inference from the conversational appropriateness of a claim to its truth is any more reliable than an inference from the conversational inappropriateness of a claim to its falsity. So DeRose's first argument for a truth/falsity asymmetry in context shifting experiments should be resisted.

DeRose's second argument, which invokes Lewis's ‘rule of accomodation’, should also be resisted. Consider the standard design of context shifting experiments, exhibited by Travis's painted leaves case and employed in Buckwalter's surveys (see Table 1, above, for a schematic representation of the standard design). The standard design invites those considering the experiment to have an intuition that what is said is *false* in one of the contexts considered. If the pressure to hear what is said as true imposed by the rule of accommodation were as strong as DeRose says it is, one would not expect the intuitions generated by traditional context shifting experiments to be as compelling as they have been. In a recent analysis of the painted leaves case, for example, Kennedy and McNally (‘Context, Content, and Compositionality’, p. 81) say:

[W]e, along with all the native speakers we have consulted, find it very difficult to deny Travis's empirical claim that [‘The leaves are green’] is false as a response to the botanist. We will therefore proceed on the assumption that denying the judgements is not an option.

And if DeRose's claim about the effect of the rule of accommodation on intuitions is correct, then redesigning Travis's painted leaves case to bring it in line with DeRose's recommendations should substantially increase the strength of the intuitions that it produces. The redesigned painted leaf case reads as follows:

A story. Pia's Japanese maple is full of russet leaves. She paints them green for a decoration. Returning, she reports, ‘That's better. The leaves are green now’. She speaks truth. A botanist friend then phones, seeking green leaves for a study of green-leaf chemistry. ‘The leaves (on my tree) aren’t green’, Pia says. Again, Pia speaks truth.

But comparing the redesigned painted leaves case with Travis's original, there is not any obvious difference in the compellingness of the intuitions elicited in the botanist context in the two different experiments (compare Tables 5 and 6):

**Table 5:** Intuitions about the Original *Painted Leaves* Case

	C1	C2
	*Decorator*	*Botanist*
‘The leaves are green’	TRUE	FALSE

**Table 6:** Intutions about the Redesigned *Painted Leaves* Case

	C1	C2
	*Decorator*	*Botanist*
‘The leaves are green’	TRUE	
‘The leaves are not green’		TRUE

Notice also that DeRose's proposed redesign involves varying both the context in which the sentence is uttered *and* the sentence that is uttered. In the standard design of context shifting experiments, the asserted sentence is held fixed and the context is varied. That allows the theorist to isolate the contribution that changing the context makes to the intuitions generated in response to the experiment. DeRose's design, in contrast, leaves it open whether it is the change in context or the change in the sentence that is responsible for the intuitions generated in response to the experiment. And, as I will discuss in the following section, there is independent reason to be suspicious of the particular change in the sentence that DeRose proposes, namely the introduction of a negative expression.

## V. Wason and True Negative Statements

A problem with DeRose's proposed modification is that there is experimental evidence indicating that a particular aspect of his design introduces a known source of error in judgement. DeRose's version of context shifting experiments involves eliciting intuitions about negative statements (‘I don’t know the bank will be open on Saturday’), and there is experimental evidence that people are less reliable at correctly judging the truth of negative statements than they are at judging affirmative statements.^29^ The psychologist P.C. Wason showed that subjects were more error-prone when asked to verify the truth of true negative statements than when they were asked to verify either true affirmative *or* false affirmative statements (even though there was no time limit on the verification task).^30^ The part of Wason's experiment most relevant for evaluating DeRose's proposal involves a verification task, in which subjects were asked to respond with ‘true’ or ‘false’ to a series of statements printed on cards about even and odd numbers, such as (9–12):

(9) Twenty-four is an even number [true affirmative].
(10) Seventy-eight is an odd number [false affirmative].
(11) Forty-six is not an odd number [true negative].
(12) Eighty-three is not an odd number [false negative].^31^

Wason observed a substantial difference between the error rate involved in verifying true negative (11.1%) and false affirmative statements (3.8%).^32^ One possible explanation for the difference in accuracy between verifying false affirmative and true negative statements is that verifying negative statements requires an act of mental conversion into their positive counterparts, which introduces more complexity and a corresponding increased possibility of error.^33^

So there is evidence that DeRose's method of setting up contextualist experiments, which involves eliciting subjects' intuitions about negative statements (‘I *don’t* know the bank will be open on Saturday’) is actually less reliable than the standard method of trying to elicit intuitions that an affirmative target sentence *TS* is true in one context and false in another, because it introduces a source of judgemental error not present in the standard design.^34^

## VI. Conclusion: Designing Context Shifting Experiments

While there are good reasons to resist DeRose's recommendation for how context shifting experiments should be modified, I am in complete agreement with the spirit of his proposal: namely, that we should pay close attention to the *design* of context shifting experiments (and the design of experiments—both thought experiments and empirical surveys—in philosophy more generally). In particular, contextualists should aim to control for known sources of *experimental bias* in the set up of their experiments. Wason's study of affirmative and negative judgements offers a clear example of one such source of bias. And that is just the beginning. There are several other known sources of experimental bias that might affect the intuitions generated by context shifting experiments.

One serious worry about context shifting experiments is that in the philosophical texts in which they are developed, they are typically accompanied by explicit statements of the contextualist's own intuitions, thereby creating a form of *experimenter bias*.^35^ There is evidence that varying the *order* in which options are presented affects intuitions about certain thought experiments, and that experiments that present *contrasting cases* to subjects generate substantially different intuitions than experiments that present subjects with single (non-contrasting) cases.^36^ Another potential source of experimental bias is the contextualist's exclusive reliance on absolute truth-value judgements, rather than allowing for the possibility of *ranking* responses to the experiments in terms of their plausibility.^37^ For the contextualist debate to rest on a secure empirical foundation, what is needed is a thorough understanding of the way subtle features of the design of context shifting experiments affect the intuitions that they generate.^38^

## Footnotes

1 See H. Cappelen and E. Lepore, *Insensitive Semantics: A Defense of Semantic Minimalism and Speech Act Pluralism* (Oxford: Blackwell, 2005), pp. 17–32.

2 Sometimes context shifting experiments refer to what a speaker *says, asserts, claims or states* by making an utterance, rather than what is said by an utterance. I will treat all of those notions as equivalent in this paper, as picking out a propositional content expressed by a speaker in making an utterance that contrasts with propositional contents that are merely *conveyed* or *implicated* by the speaker in making the utterance. For that understanding of the notion of what is said, see F. Recanati, ‘What is Said’, *Synthese*, 128 (2001), pp. 75–91. I am taking the generic grouping of ‘what is asserted, claimed or stated’ from R. Stainton, ‘Contextualism in Epistemology and the Context-Sensitivity of “Knows”’, in J.K. Campbell, M. O’Rourke and H.S. Silverstein (eds), *Knowledge and Skepticism*, (MIT Press, 2010), pp. 113–39.

3 This additional remark is from the version of the experiment that appears in C. Travis, ‘On Constraints of Generality’, *Proceedings of the Aristotelian Society*, 94 (1994), pp. 165–88, at p. 172.

4 The example is from C. Travis, ‘Pragmatics’, in B. Hale and C. Wright (eds), *A Companion to the Philosophy of Language* (Oxford: Blackwell, 1997), pp. 87–107, at p. 89.

5 See, for example, N. Hansen, ‘Color Adjectives and Radical Contextualism’, *Linguistics and Philosophy*, 34 (2011), pp. 201–21; C. Kennedy and L. McNally, ‘Color, Context and Compositionality’, *Synthese*, 174 (2010), pp. 79–98; S. Predelli, ‘Painted Leaves, Context, and Semantic Analysis’, *Linguistics and Philosophy*, 28 (2005), pp. 351–74; D. Rothschild and G. Segal, ‘Indexical Predicates’, *Mind & Language*, 24 (2009), pp. 467–93; M. Sainsbury, ‘Two Ways to Smoke a Cigarette’, *Ratio*, 14 (2001), pp. 386–406; Z.G. Szabó, *Problems of Compositionality*, (New York: Garland, 2000) and ‘Adjectives in Context’, in I. Kenesei and R.M. Harnish (eds), *Perspectives on Semantics, Pragmatics, and Discourse* (Amsterdam: John Benjamins, 2001), pp. 119–46; and Travis, ‘On Contraints of Generality’ and ‘Pragmatics’.

6 Examples of recent work that considers the methods by which intuitions are elicited includes A. Pinillos, ‘Knowledge, Experiments, and Practical Interests’, in J. Brown and M. Gerken (eds.), *New Essays on Knowledge Ascriptions* (Oxford UP, forthcoming), W. Buckwalter, ‘Non-Traditional Factors in Judgments about Knowledge’, *Philosophy Compass*, 7 (2012), pp. 278–289), and P. Rysiew, ‘Surveys, Intuitions, Knowledge Attributions’, *Philosophical Studies*, 156 (2011), pp. 111–20.

7 See K. DeRose, ‘The Ordinary Language Basis for Contextualism, and the New Invariantism’, *The Philosophical Quarterly*, 55 (2005), pp. 172–98, *The Case for Contextualism* (Oxford UP, 2009), and ‘Contextualism, Contrastivism and X-Phi Surveys’, *Philosophical Studies*, 156 (2011), pp. 81–110.

8 The bank cases appear in K. DeRose, ‘Contextualism and Knowledge Attributions’, *Philosophy and Phenomenological Research*, 52 (1992), pp. 913–29, at pp. 913–14, and in *The Case for Contextualism*, pp. 1–2.

9 DeRose, ‘Contextualism and Knowledge Attributions’, p. 914.

10 DeRose, ‘The Ordinary Language Basis for Contextualism, and the New Invariantism’, p. 173.

11 ‘The Ordinary Language Basis for Contextualism, and the New Invariantism’, p. 174, and *The Case for Contextualism*, p. 51.

12 ‘The Ordinary Language Basis for Contextualism, and the New Invariantism’, p. 174, and *The Case for Contextualism*, p. 52.

13 DeRose is quoting D. Lewis, ‘Causation as Influence’, *The Journal of Philosophy*, 97 (2000), pp. 182–197, at p. 196.

14 ‘The Ordinary Language Basis of Contextualism and the New Invariantism’, pp. 174–175, and *The Case for Contextualism*, p. 52.

15 DeRose also criticises these studies for neglecting other important aspects of the proper design of the relevant experiments—see his ‘Contextualism, Contrastivism, and X-Phi Surveys’ for discussion.

16 See W. Buckwalter, ‘Knowledge Isn’t Closed on Saturdays’, *Review of Philosophy and Psychology*, 1 (2010), pp. 395–406.

17 ‘Knowledge Isn’t Closed on Saturdays’, p. 401.

18 For theoretical and empirical challenges to Buckwalter's conclusion, see N. Hansen and E. Chemla, ‘Experimenting on Contextualism’, *Mind & Language*, (forthcoming).

19 See DeRose, ‘Contextualism, Contrastivism, and X-Phi Surveys, p. 87. DeRose also criticises Buckwalter's findings on the grounds that he alters DeRose's bank cases in order to isolate the effects of changing stakes and what possibilities of error are salient. I will not discuss this criticism here; I am interested in the way DeRose deploys the truth/falsity asymmetry in his response to Buckwalter.

20 See Recanati, ‘What is Said’ and ‘Literalism and Contextualism: Some Varieties’, in G. Preyer and G. Peter (eds), *Contextualism in Philosophy: Knowledge, Meaning, Truth* (Oxford UP, 2005), pp. 171–96, at p. 177. I am adopting the use of ‘minimalism’ and ‘free enrichment’ from Recanati.

21 From K. Bach, ‘Semantic Slack’, in S.L. Tsohatzidis (ed), *Foundations of Speech Act Theory* (London: Routledge, 1994), pp. 267–91, at p. 267.

22 See, for example, the discussion of the example in F. Recanati, *Literal Meaning* (Cambridge UP, 2004), p. 74.

23 Example (2) is from P. Lasersohn, ‘Pragmatic Halos’, *Language*, 75 (1999), pp. 522–51, at p. 522.

24 Example (3) is from D. Sperber and D. Wilson, ‘Loose Talk’, *Proceedings of the Aristotelian Society*, 86 (1985), pp. 153–71, at p. 163.

25 Examples (4–7) are from D. Wilson and D. Sperber, ‘Truth and Relevance’, *Mind*, 111 (2002), at p. 592.

26 Example (8) is inspired by P. Unger, *Ignorance* (Oxford UP, 1975), pp. 51–2.

27 See Stainton, ‘Contextualism in Epistemology and the Context-Sensitivity of “Knows”’ for a discussion of the relevance of free enrichment to the debate over epistemic contextualism.

28 Thanks to Paul Égré and an anonymous reviewer for emphasising the relevance of different theories of what is said to the plausibility of DeRose's presumption of truth for conversationally appropriate claims.

29 In ‘Contextualism, Contrastivism, and X-Phi Surveys’ (p. 94 n. 18), DeRose recognizes that ‘Some of the potential problems [with giving DeRose's bank cases to survey takers] may come from there being a negation inside the claim being evaluated in HIGH’. But he doesn’t say why it might be a problem.

30 P.C. Wason, ‘Response to Affirmative and Negative Binary Statements’, *British Journal of Psychology*, 52 (1961), pp. 133–42.

31 ‘Response to Affirmative and Negative Binary Statements’, p. 134.

32 ‘Response to Affirmative and Negative Binary Statements’, p. 137. Wason only presents raw data in his paper (32/288 errors on true negative verification tasks and 11/288 errors on false affirmatives). It is difficult to run appropriate statistical tests on this data, because Wason does not present his results subject by subject. Thanks to Emmanuel Chemla for discussion of this issue.

33 See L. Horn, *A Natural History of Negation*, (Stanford: CSLI Press, 2001), at p. 170; B. Geurts, ‘Donkey Business’, *Linguistics and Philosophy*, 25 (2001), pp. 129–56, at pp. 152–53.

34 Another relevant consideration when considering whether to include negation in the design of experiments is the role that negation plays in triggering ‘system 2’ processing. For discussion, see J. Nagel, ‘The Psychological Basis of the Harman-Vogel Paradox’, *Philosophers’ Imprint*, 11 (2011), pp. 1–28. Thanks to Mikkel Gerken for pointing this out.

35 For an overview of types of experimenter bias, see R. Rosenthal, *Experimenter Effects in Behavioral Research*, (New York: Irvington, 1976). Some participants in debates about contextualism and related theories have claimed that experimenter bias affects intuitions in certain cases: J. Schaffer, ‘The Irrelevance of the Subject’, *Philosophical Studies*, 127 (2006), pp. 87–107, at p. 88, recommends modifying the version of DeRose's bank cases that appears in J. Stanley, *Knowledge and Practical Interests*, (Oxford UP, 2005), to eliminate the effects of experimenter bias, and Z.G. Szabó, in ‘Sensitivity Training’, *Mind & Language*, 21 (2006), pp. 31–8, at p. 37 n. 19, worries that intuitions generated by one of the context shifting experiments employed by Cappelen and Lepore in their *Insensitive Semantics* are unreliable ‘because [the thought experiment] starts with dogmatic claims that distort the reader's subsequent intuitions’.

36 For discussion of ordering effects, see J. May, W. Sinnott-Armstrong, J.G. Hull and A. Zimmerman, ‘Practical Interests, Relevant Alternatives, and Knowledge Attributions: An Empirical Study’, *Review of Philosophy and Psychology*, 1 (2010), pp. 265–73; E. Schwitzgebel and F. Cushman, ‘Expertise in Moral Reasoning? Order Effects on Moral Judgments in Professional Philosophers and Non-Philosophers’, *Mind & Language*, 27 (2012); and S. Swain, J. Alexander and J. Weinberg, ‘The Instability of Philosophical Intuitions: Running Hot and Cold on Truetemp’, *Philosophy and Phenomenological Research*, 76 (2008), pp. 138–55. For discussion of contrast effects, see M. Phelan, ‘Evidence that Stakes Don’t Matter for Evidence’, unpublished manuscript.

37 For discussion of the possibility of ranking responses to thought experiments, see P. Unger, ‘Toward a Psychology of Common Sense’, *American Philosophical Quarterly*, 19 (1982), pp. 117–29.

38 Thanks to Paul Égré, Mikkel Gerken, Emmanuel Chemla, Ben Chan, Jason Bridges, Martin Gustafsson, Chauncey Maher, Eliot Michaelson, Zed Adams, members of the Doc’in Nicod workshop at the Institut Jean-Nicod, audience members at Åbo Akademi, and the anonymous reviewers for this journal for their helpful comments on the contents of this paper. The research leading to these results has received funding from the European Research Council under the European Community's Seventh Framework Programme (FP7/2007-2013) / ERC grant agreement no. 229 441 – CCC.

